# Delayed postoperative radiation therapy in local control of squamous cell carcinoma of the tongue and floor of the mouth

**DOI:** 10.1590/S1679-45082014AO3006

**Published:** 2014

**Authors:** Ali Amar, Helma Maria Chedid, Otávio Alberto Curioni, Rogério Aparecido Dedivitis, Abrão Rapoport, Claudio Roberto Cernea, Lenine Garcia Brandão

**Affiliations:** 1 Hospital Heliópolis, São Paulo, SP, Brazil.; 2 Universidade de São Paulo, São Paulo, SP, Brazil.

**Keywords:** Carcinoma, squamous cell/radiotherapy, Tongue neoplasms/radiotherapy, Mouth neoplasms/radiotherapy, Time factors, Recurrence

## Abstract

**Objective:**

To evaluate the effect of time between surgery and postoperative radiation therapy on local recurrence of squamous cell carcinoma of the tongue and floor of the mouth.

**Methods:**

A total of 154 patients treated between 1996 and 2007 were selected considering local recurrence rate and time of the adjuvant radiotherapy.

**Results:**

Local recurrence was diagnosed in 54 (35%) patients. Radiation therapy reduced the rate of local recurrences, although with no statistical significance. The time between surgery and initiation of postoperative radiotherapy did not significantly influence the risk of local recurrence in patients referred to adjuvant treatment (p=0.49).

**Conclusion:**

In the presence of risk factors for local recurrence, a short delay in starting the adjuvant radiation therapy does not contraindicate its performance.

## INTRODUCTION

Usually, postoperative radiation therapy for patients with head and neck cancer is indicated when there is a high risk of recurrence. Ideally, radiation therapy should begin 6 weeks after surgical treatment. Delaying the start of adjuvant radiation therapy decreases its therapeutic effect due to hypoxia of the scarring tissue and repopulation with tumor cells.^(1,2)^ This is the reason why many studies tried to investigate the use of preoperative radiotherapy in 1960s and 1970s. In spite of the evidence provided by experimental studies, clinical trials have not shown that patients with malignant head and neck tumors stand to benefit from this therapeutical approach.^(3)^ Some authors believe that the total time, including waiting and treatment, is more important than the wait alone, and that a delay could be compensated by accelerated radiation therapy regimens.^(4-6)^ However, it still remains unclear if patients who are unable to start radiation therapy within the ideal timeframe can still benefit from late adjuvant treatment.

## OBJECTIVE

To assess the effect of delayed radiation therapy in the local control of squamous cell carcinoma of the tongue and floor of the mouth.

## METHODS

The medical records of 184 patients with squamous cell carcinoma of the tongue and floor of the mouth (staging II to IV) we studied the patients underwent surgery between January 1996 and December 2007 and were treated at the Head and Neck and ENT Surgical Unit of the *Hospital* *Heliópolis* in the capital city of São Paulo. Thirty-one cases were excluded because the follow-up period was under 12 months; therefore, 153 patients met the inclusion criteria. As far as sex is concerned, 134 were male and the mean age was 52 years (range of 22 to 78 years). The local recurrence rate was investigated in relation to the time of postoperative radiation therapy, and the subjects were divided into three groups: less than 6 weeks after surgery, between 6 to 8 weeks after surgery and more than 8 weeks after surgery.

The criteria for indication of postoperative radiation therapy were heterogeneous, and certain patients failed to complete the treatment proposed. For analysis, only pT3 or pT4 staging and compromised or narrow surgical margins were considered as an indication for radiotherapy. When performed due to regional disease, radiation therapy was deemed as having no indication.

The statistical analysis used the χ^2^ test with the Yates correction, and only differences with p<0.05 were considered statistically significant.

The present study was approved by the IRB (Internal Research Board) of the *Hospital Heliópolis* under number 271/2004.

## RESULTS

Fifty-four patients (35%) presented local recurrence. While 74 patients received postoperative radiation therapy at the conventional fractioning and an average dose of 60Gy (41 to 72), 58 patients (78%) received a dose ≥60Gy. Postoperative radiation therapy was initiated between 2 to 18 weeks after surgery.

Radiotherapy reduced the rate of local recurrence from 61% to 40% in those cases this modality had been indicated (p=0.07), and a trend towards statistical significance was observed. The rate of local recurrence in irradiated patients with a positive margin fell from 66% to 48% (p=0.47), and from 60% to 42% in patients who were pT3 or pT4 (p=0.19), although with not statistical significance.

Among the patients with an indication for adjuvant treatment, the time between surgery and postoperative radiotherapy did not affect local recurrence rate, and the result was not statistically significant (p=0.49) as seen in table 1.

**Table 1:** Local recurrence according to indication and time of radiation therapy

Indication for RT	Time of RT (weeks)	Local recurrence		Total
		Yes	No	
**n (%)**	**n (%)**
Yes	No	21 (61)	13 (39)	34
	<6	11 (44)	14 (56)	25
	6-8	5 (45)	6 (55)	11
	>8	6 (31)	13 (69)	19
No	No	9 (18)	39 (82)	48
	<6	1 (11)	8 (89)	9
	6-8	0	1 (100)	1
	>8	1 (20)	4 (80)	5

Total		54 (35)	99 (65)	153

RT: postoperative radiation therapy.

## DISCUSSION

This study showed a lower local recurrence rate in patients who received postoperative radiation therapy, even after 6 weeks, albeit with no statistical significance. It may be the case that this delay did not significantly affect the outcome because regional recurrence was not an inclusion criterion. Another reason may be that, because cases with a poorer prognosis are required to delay starting adjuvant therapy due to postoperative complications, this was a rather homogeneous series in terms of primary site. Moreover, the higher doses that are currently used seem to compensate for this delay. In face of the existing literature, every possible effort should be made to start postoperative radiation therapy as early as possible.^(2-8)^ Although no time limit has been established, it is known that when facing recurrence, the exclusive use of radiation therapy can be disappointing. Therefore, there must be a moment in time as of which postoperative radiation therapy contributes very little to disease control.^(9,10)^ It has been suggested that 6 weeks be the time limit established to achieve local control, provided that the dose is lower than 60Gy.^(1)^ This is what we abide by at our practice; however, in 36 patients (23.5%), this was not the case, either because of the patient´s adverse conditions (dehiscences and fistulas) or because of certain practical limitations typical of a public hospital.

In the presence of several aggressiveness criteria or, more specifically, in cases which the diagnosis of recurrence is more difficult, such as in major reconstructions or when the resection borders make a second surgery unlikely, the use of radiation therapy is justified, even if later. However, it is precisely among patients with more risk factors that the delay in initiating radiation therapy seems to have the strongest impact in terms of locoregional recurrence.^(8)^ When treating mouth cancer, local recurrence remains an important reason for failure, but radiotherapy may also make it more difficult to diagnose recurrence, as well as to reduce the feasibility of performing a salvage surgery.^(10)^ Postoperative radiation therapy targets those fields that encompass the entire area under treatment, including both the bed of the primary site, as well as neck dissection areas, even when adjuvant treatment is indicated for one site only.

This study did not consider the indication of radiotherapy to treat neck metastases alone, since many of them were associated with recurrence at the primary site. Given the shortage of resources, the delay of adjuvant therapy has become a current issue and, unfortunately, in the case of squamous cell carcinoma of the upper air and digestive tracts, the efficacy of late radiation therapy has not been satisfactorily investigated.

## CONCLUSION

In the presence of risk factors for local recurrence, a short delay in starting adjuvant radiotherapy does not represent a contraindication against this line of treatment. When postoperative radiotherapy was performed, a reduction in local recurrence, as well as in cases with positive margin and pT3 or pT4 was seen albeit with no statistical significance.
