# Supplementary material for: Delayed postoperative radiation therapy in local control of squamous cell carcinoma of the tongue and floor of the mouth
Source: Einstein (Sao Paulo). 2014 Oct-Dec;12(4):477–9. doi: 10.1590/S1679-45082014AO3006 (PMC4879915; doi:10.1590/S1679-45082014AO3006)
Supplement: Translation

# Translation: Retardo da radioterapia pós-operatória no controle local do carcinoma epidermoide de língua e soalho de boca

**Authors:** Ali Amar, Helma Maria Chedid, Otávio Alberto Curioni, Rogério Aparecido Dedivitis, Abrão Rapoport, Claudio Roberto Cernea, Lenine Garcia Brandão

**Affiliations:** 1 Hospital Heliópolis, São Paulo, SP, Brasil.; 2 Universidade de São Paulo, São Paulo, SP, Brasil.

**Keywords:** Neoplasias de células escamosas/radioterapia, Neoplasias da língua/radioterapia, Neoplasias bucais/radioterapia, Fatores de tempo, Recidiva

## Abstract

**Objetivo:**

Avaliar o efeito do tempo entre o tratamento cirúrgico e a radioterapia pós-operatória nas recidivas locais do carcinoma epidermoide de língua e soalho da boca.

**Métodos:**

Foram selecionados 154 pacientes tratados entre 1996 e 2007, considerando a frequência das recidivas locais e o tempo para início da radioterapia adjuvante.

**Resultados:**

As recidivas locais foram diagnosticadas em 54 (35%) pacientes. A radioterapia reduziu a frequência de recidivas locais, embora sem significância estatística. O tempo entre a cirurgia e o início da radioterapia pós-operatória não influenciaram, de forma significante, no risco de recidivas locais entre o pacientes que tinham indicação de tratamento adjuvante (p=0,49).

**Conclusão:**

Na presença de fatores de risco para recidiva local, um pequeno atraso no início da radioterapia adjuvante não contra-indica sua realização.

## INTRODUÇÃO

A radioterapia pós-operatória em pacientes portadores de câncer de cabeça e pescoço é usualmente empregada nos casos com alto risco de recidiva e, idealmente, iniciada até 6 semanas após o tratamento cirúrgico. O atraso da radioterapia adjuvante acarreta um menor efeito terapêutico, atribuído à hipóxia do tecido cicatricial e à repopulação celular tumoral.^(1,2)^ Por esse motivo, a radioterapia pré-operatória foi explorada em diversos estudos nas décadas de 1960 e 1970. Apesar das evidências encontradas em estudos experimentais, os ensaios clínicos não mostraram benefícios dessa prática nos tumores malignos da cabeça e pescoço.^(3)^ Alguns autores consideram que o tempo total, incluindo a espera e o tratamento, seja mais importante do que o tempo de espera isoladamente, com um eventual atraso sendo compensado com esquemas acelerados de radioterapia.^(4-6)^ Contudo, ainda não está definido se pacientes que não conseguem iniciar a radioterapia no momento ideal ainda se beneficiam de um tratamento adjuvante tardio.

## OBJETIVO

Avaliar o efeito do atraso da radioterapia no controle local do carcinoma epidermoide de língua e soalho da boca.

## MÉTODOS

Foram avaliados os prontuários de 184 pacientes com carcinoma epidermoide de língua e soalho oral em estádios II a IV, operados entre janeiro de 1996 e dezembro de 2007, tratados no Serviço de Cirurgia de Cabeça e Pescoço e Otorrinolaringologia do Hospital Heliópolis, na cidade de São Paulo (SP). Foram excluídos 31 casos com menos de 12 meses de seguimento; assim, foram avaliados 153 pacientes. Quanto ao sexo, 134 eram masculinos. A média da idade foi de 52 anos (variação de 22 a 78 anos). Foi aferida a frequência de recidiva local de acordo com o momento da realização de radioterapia pós-operatória, sendo dividida em três categorias: antes de 6 semanas, de 6 a 8 semanas e mais que 8 semanas da cirurgia.

Os critérios de indicação de radioterapia pós-operatória foram heterogêneos no período de estudo, assim como alguns pacientes também não realizaram o tratamento proposto. Para análise, foram considerados indicações de radioterapia o estadiamento pT3 ou pT4, e margens cirúrgicas comprometidas ou exíguas. Quando realizada por causa da doença regional, foi considerada sem indicação.

A análise estatística usou o teste χ^2^ com correção de Yates, considerando significantes as diferenças com valor de p<0,05.

O presente estudo foi aprovado pelo Comitê de Ética em Pesquisa do Hospital Heliópolis, sob o número 271/2004.

## RESULTADOS

As recidivas locais foram diagnosticadas em 54 (35%) pacientes. A radioterapia pós-operatória foi realizada em 74 casos, com fracionamento convencional e dose média de 60Gy (41 a 72), sendo que 58 pacientes (78%) receberam dose ≥60Gy. A radioterapia pós-operatória foi iniciada de 2 a 18 semanas após a cirurgia.

A radioterapia reduziu a frequência de recidivas locais de 61 para 40% nos casos em que se atribuiu indicação de radioterapia pós-operatória (p=0,07), notando-se uma tendência a significância estatística. A frequência de recidiva local nos casos irradiados caiu de 66 para 48% nos casos com margem positiva (p=0,47) e de 60 para 42% nos casos pT3 ou pT4 (p=0,19), porém sem significado estatístico.

O tempo entre a cirurgia e a radioterapia pós-operatória não influenciou a frequência de recidivas locais entre os pacientes que tinham indicação de tratamento adjuvante, não tendo sido estatisticamente significativo (p=0,49), conforme demonstrado na tabela 1.

**Tabela 1:** Recidivas locais, de acordo com a indicação e momento da radioterapia

Indicação RT	Momento RT (semanas)	Recidiva local		Total
		Sim	Não	
		n (%)	n (%)	
Sim	Não	21 (61)	13 (39)	34
	<6	11 (44)	14 (56)	25
	6-8	5 (45)	6 (55)	11
	>8	6 (31)	13 (69)	19
Não	Não	9 (18)	39 (82)	48
	<6	1 (11)	8 (89)	9
	6-8	0	1 (100)	1
	>8	1 (20)	4 (80)	5

Total		54 (35)	99 (65)	153

RT: radioterapia pós-operatória.

## DISCUSSÃO

No presente estudo, observamos menor frequência de recidivas locais com a realização da radioterapia pós-operatória, mesmo quando realizada após 6 semanas, embora sem significância estatística. Talvez o atraso não tenha tido repercussão significativa, por não terem sido incluídas as recidivas regionais. Outro motivo também está na casuística homogênea em relação ao sítio primário, uma vez que alguns casos com pior prognóstico sofrem atraso no tratamento adjuvante relacionado às complicações pós-operatórias. Adicionalmente, as doses mais altas empregadas atualmente parecem compensar o atraso no início do tratamento. Diante da literatura existente, deve ser feito todo o esforço possível para que a radioterapia pós-operatória seja realizada precocemente.^(2-8)^ Um tempo limite ainda não foi estabelecido, mas sabemos que, diante de uma recidiva, o tratamento radioterápico exclusivo tem resultados desapontadores e, portanto, deve haver um momento em que a radioterapia pós-operatória acrescente pouquíssimo no controle da doença.^(9,10)^Sugere-se o tempo de 6 semanas como limítrofe para o controle local desde que com dose abaixo de 60Gy,^(1)^orientação que procuramos seguir em nossa prática; contudo, em 36 pacientes (23,5%), isso não foi factível, seja devido a condições adversas dos pacientes (deiscências e fístulas), seja por limitações encontradas dentro da rotina de um hospital público.

Na presença de múltiplos critérios de agressividade ou, mais especificamente, nas situações em que o diagnóstico de uma recidiva pode ser dificultado, como nas grandes reconstruções ou, ainda, quando os limites da ressecção tornam improvável uma nova cirurgia, é justificável realizar a radioterapia, mesmo que tardiamente. Contudo, justamente entre os pacientes com mais fatores de risco, o atraso no início da radioterapia parece ter maior impacto nas recidivas loco-regionais.^(8)^ As recidivas locais ainda são uma causa importante de falha no tratamento do câncer da boca, mas a radioterapia também pode dificultar o diagnóstico precoce de uma recidiva, assim como também reduz a possibilidade de resgate cirúrgico.^(10)^ A radioterapia pós-operatória ainda emprega campos que abrangem toda a área de tratamento, incluindo tanto o leito do sítio primário como as áreas de drenagem cervical, mesmo que a indicação do tratamento adjuvante esteja em uma única localização.

O presente estudo não considerou a indicação da radioterapia no tratamento das metástases cervicais isoladamente, uma vez que muitas destas estivessem associadas com recidiva no sítio primário. Diante da escassez de recursos, a possibilidade de atraso no tratamento adjuvante é uma questão atual e, infelizmente, a eficácia da radioterapia iniciada tardiamente ainda não foi avaliada de forma satisfatória nos carcinomas epidermoides das vias aerodigestivas superiores.

## CONCLUSÃO

Na presença de fatores de risco para recidiva local, um pequeno atraso no início da radioterapia adjuvante não contraindica sua realização. Houve redução das recidivas locais quando se realizou radioterapia pós-operatória, bem como nos casos com margem positiva e nos casos pT3 ou pT4, porém, sem significado estatístico.
